# A Metabolome and Microbiome Analysis of Acute Myeloid Leukemia: Insights into the Carnosine–Histidine Metabolic Pathway

**DOI:** 10.3390/toxics12010014

**Published:** 2023-12-22

**Authors:** Binxiong Wu, Yuntian Xu, Miaomiao Tang, Yingtong Jiang, Ting Zhang, Lei Huang, Shuyang Wang, Yanhui Hu, Kun Zhou, Xiaoling Zhang, Minjian Chen

**Affiliations:** 1 Department of Hygienic Analysis and Detection, School of Public Health, Nanjing Medical University, Nanjing 211166, China; wubinxiong@stu.njmu.edu.cn; 2 State Key Laboratory of Reproductive Medicine and Offspring Health, Center for Global Health, School of Public Health, Nanjing Medical University, Nanjing 211166, China; uu11ut@163.com (Y.X.); tmm_2023@163.com (M.T.); ytjiang61@163.com (Y.J.); 18756555623@163.com (L.H.); wsy8032@163.com (S.W.); zk@njmu.edu.cn (K.Z.); 3 Key Laboratory of Modern Toxicology of Ministry of Education, School of Public Health, Nanjing Medical University, Nanjing 211166, China; 4 Women’s Hospital of Jiangnan University, Wuxi 214002, China; zhangting@njmu.edu.cn; 5 Sir Run Run Hospital of Nanjing Medical University, Nanjing 211166, China; njjshyh@126.com; 6 Department of Epidemiology, Center for Global Health, School of Public Health, Nanjing Medical University, Nanjing 211166, China

**Keywords:** acute myeloid leukemia, gut microbiota, metabolomics, metabolic pathway

## Abstract

Metabolism underlies the pathogenesis of acute myeloid leukemia (AML) and can be influenced by gut microbiota. However, the specific metabolic changes in different tissues and the role of gut microbiota in AML remain unclear. In this study, we analyzed the metabolome differences in blood samples from patients with AML and healthy controls using UPLC-Q-Exactive. Additionally, we examined the serum, liver, and fecal metabolome of AML model mice and control mice using UPLC-Q-Exactive. The gut microbiota of the mice were analyzed using 16S rRNA sequencing. Our UPLC-MS analysis revealed significant differences in metabolites between the AML and control groups in multiple tissue samples. Through cross-species validation in humans and animals, as well as reverse validation of Celastrol, we discovered that the Carnosine–Histidine metabolic pathway may play a potential role in the occurrence and progression of AML. Furthermore, our analysis of gut microbiota showed no significant diversity changes, but we observed a significant negative correlation between the key metabolite Carnosine and *Peptococcaceae* and *Campylobacteraceae*. In conclusion, the Carnosine–Histidine metabolic pathway influences the occurrence and progression of AML, while the gut microbiota might play a role in this process.

## 1. Introduction

Acute myeloid leukemia (AML) is a malignant tumor characterized by abnormal proliferation, differentiation disorders, and blocked apoptosis of myeloid stem cells. The accumulation of leukemia cells causes damage by inhibiting normal hematopoiesis and infiltrating other tissues and organs. The prognosis of the disease is poor, with an average survival period of only about 3 months without specific treatment, and the 5-year survival rate after treatment is only about 10–35% [1]. For decades, chemotherapy has remained the main treatment for AML [2], with the goal of inducing remission. However, elderly patients, who are the main population affected by the disease, often have difficulty tolerating chemotherapy [3]. In addition, relapse is difficult to avoid, and the survival rate drops sharply after relapse. AML is highly heterogeneous, and there has been little progress in treatment methods for a long time. Therefore, a better understanding of the development and progression mechanisms of AML is required in order to effectively prevent the disease and mitigate its incidence.

Metabolomics is the scientific study of the metabolite profile of an organism under specific physiological or pathological conditions. By analyzing the types and quantities of metabolites in an organism, it can reveal the metabolic pathways, regulatory mechanisms, and associations with disease development. Metabolic changes may play an important role in the occurrence and development of AML. In AML research, metabolomics techniques have been widely used to analyze the blood metabolism of groups of patients with AML and controls, in order to discover metabolic pathways and molecular biomarkers associated with AML [4], and to aid in disease diagnoses and outcome prediction [5,6]. However, organismal metabolism is a complex system involving multiple metabolic pathways and molecular interactions. Single-type samples may not fully reflect the overall changes in organismal metabolism. Studies have found that plasma has better reproducibility, while serum has higher sensitivity [7,8]. Using different sample types can lead to more comprehensive results. Therefore, in this study, we analyzed the metabolic changes in serum, liver, and fecal samples of mice, as well as serum and plasma samples from human subjects to complement each other and to enhance population metabolic information, for validation and replication. Additionally, in the mouse experiments, we used a natural compound, Celastrol, which has been shown to possess anti-leukemia activity, as a reversing agent [9], to further validate key metabolites.

The gut microbiota, a crucial microbial component residing in the gastrointestinal tract, have been found to play a significant role in metabolism and immunity due to their large numbers and diverse genomes. *Helicobacter pylori*, through chronic inflammation and specific virulence factors, has emerged as a major risk factor for gastric cancer [10]. Similarly, *Fusobacterium nucleatum* has been implicated in inducing colorectal cancer metastasis by downregulating m6A gene modification [11]. It has been reported that the gut microbiota can regulate and maintain normal hematopoiesis [12]. In the research of AML, researchers have discovered that the composition of the gut microbiota is related to the treatment [13]. Currently, fecal microbiota transplantation (FMT) is being used in clinical practice to correct gut dysbiosis and eradicate multidrug-resistant bacteria, thereby treating diseases. These findings suggest that the gut microbiota may also play a role in the occurrence and development of AML. Therefore, it is necessary to explore the complex relationship between the gut microbiota and the development of AML.

The gut microbiota can influence the human body by producing metabolites, with short-chain fatty acids (SCFAs) [14] and tryptophan [15] being among the most extensively studied. These metabolites help maintain gut barrier integrity and reduce disease-related effects. Feces consist of undigested food residues, bacterial metabolites, and other waste materials from the intestines, making changes in fecal metabolites reflective of the metabolic activities of the gut microbiota. By analyzing 16S rRNA in feces, we can gain insights into the composition and function of the gut microbiota, and further investigate the relationship between the gut microbiota and human health. The regulation of organismal metabolism by the gut microbiota genome plays a crucial role in normal physiological functions and disease responses. However, there is still a lack of research on microbiota-dependent metabolites in AML. Additionally, the gut microbiota are susceptible to influences from the diet [16], medications [17], and the environment, making it challenging to conduct population studies with limited confounding variables. Therefore, we aimed to elucidate the alterations in the gut microbiota in the context of AML using a mouse model, which provides a relatively stable and controlled environment. Additionally, we sought to investigate the relationship between changes in the gut microbiota and metabolic alterations.

This study aimed to investigate the non-targeted metabolic changes in multiple samples of AML using a mouse model and blood samples from patients with AML, employing UPLC-MS technology. Additionally, a reversal experiment was conducted using an AML inhibitor, Celastrol, to elucidate key metabolic processes. Furthermore, changes in the gut microbiota were revealed through 16S rRNA amplicon sequencing, and the potential mechanisms of gut microbiota in AML metabolic changes were inferred through statistical and biological correlations. The significance of this study lies in the establishment of a mouse model and the utilization of various techniques to uncover the overall metabolic changes in multiple samples and across species in AML, as well as exploring the potential connections between these changes and the gut microbiota. This research is important for a deeper understanding of the pathogenesis of AML, and identification of new prevention and treatment targets.

## 2. Materials and Methods

### 2.1. AML Mouse Model

SPF-grade BALB/c nude mice (male, 5 weeks old, weighing 18–20 g) were purchased from Shanghai Lingchang Biotechnology Co., Ltd. (Shanghai, China). and housed at the Animal Experimental Center of Nanjing Medical University. The mice were kept under constant environmental conditions with a humidity of 55 ± 10% and a temperature of 22 ± 2 °C, with a 12 h light/dark cycle. They had unrestricted access to water and food. The principles of the 3Rs were followed to ensure the welfare of the experimental animals. After one week of adaptation, the mice were randomly divided into a control group (*n* = 8) and a treatment group (*n* = 25). There was no statistically significant difference of weight between groups (Figure S1). HL-60 cells in the logarithmic growth phase were resuspended in PBS at a density of 5 × 10^7^ cells/mL and subcutaneously injected into the right dorsal axillary region of the treatment group mice, with an injection volume of 100 µL. The control group mice were injected with an equal volume of physiological saline. When the tumor volume reached 200–250 mm^3^, the mice were randomly divided into the AML group (*n* = 8), low-dose Celastrol treatment group (*n* = 8), and high-dose Celastrol treatment group (*n* = 9). The Celastrol treatment groups were intraperitoneally injected with 200 µL of a solution containing Celastrol at concentrations of 0.1 mg/mL and 0.2 mg/mL, respectively, once daily. Previous research has demonstrated the inhibitory efficacy of Celastrol against AML at these specific concentrations [18,19]. The control group and AML group were given an equal volume of physiological saline daily. When the tumor volume of the AML group mice reached 2500–3000 mm^3^, samples were collected. Adequate fecal samples were collected from the intestines of the dissected mice and stored in clean containers for 16S rRNA gene sequencing. Various organs were also collected and stored in liquid nitrogen for the subsequent analysis.

### 2.2. Study Population

This section describes a cross-sectional study that included two groups: an AML group (*n* = 19) diagnosed between October 2020 and December 2020 and a recruited healthy control group (*n* = 35) at Jiangsu Provincial Hospital and Nanyang First People’s Hospital. The inclusion criteria for the AML group were as follows: diagnosed with AML based on blood cell examination with no liver or kidney diseases, and no recent use of medications that could affect metabolism. The inclusion criteria for the healthy control group were the exclusion of individuals with common endocrine disorders, acute or chronic gastroenteritis, severe impairment of cardiac, hepatic, pulmonary, or renal structure and function, and individuals with tumors. Basic information, including age, gender, and medical history, was recorded for all participants for the subsequent analysis. Fasting blood samples were collected in the morning after an 8–12 h fast (plasma group: *n* = 30, serum group: *n* = 24) and stored at −80 °C for the subsequent UPLC-MS analysis. This study was conducted in accordance with the ethical standards of Jiangsu Provincial Hospital and Nanyang First People’s Hospital. All participants involved in this study provided informed consent.

### 2.3. Metabolomic Analysis

The metabolomic analysis of plasma, serum, liver, and feces was performed using the UPLC-Q-Exactive platform (UPLC Ultimate 3000 system, Dionex, Germering, GER; Q-Exactive Orbitrap, Thermo Fisher Scientific, Bremen, GER) according to our previous report [20]. Briefly, protein precipitation was conducted with a methanol–water mixture containing isotope-labeled internal standards. The fecal samples were homogenized using vortexing and ultrasonic wave treatment as described in our previous study [21]. The supernatant was concentrated to dryness using a centrifugal concentrator (Labconco, Kansas, MO, USA). The dried sample was reconstituted and ready for the analysis. A Hypersil GOLD C18 column (100 mm × 2.1 mm, 1.9 μm, Thermo Fisher Scientific, Vilnius, Lithuania) was used for a chromatography system. Acetonitrile containing 0.1% formic acid was used as mobile phase A and ultrapure water containing 0.1% formic acid was used as mobile phase B. The flow rate was set at 0.40 mL/min (column temperature at 40 °C). The ionization mode for mass spectrometry was heated electrospray ionization (HESI). The spray voltage was set at 3.5 kV for positive ion mode and 2.5 kV for negative ion mode. Full scan mode was used with a scan range of 70 to 1050 *m*/*z* and a resolution of 70,000. The accurate mass and retention time of metabolites were compared with those of metabolite standards using a self-built standard library using TraceFinder 3.1 software for metabolite identification. All samples were analyzed in a randomized fashion to avoid bias of the injection order. The data were further analyzed after integral normalization [22]. An equivalent volume from each sample under investigation was combined to create quality control (QC) samples, and a blank solvent was utilized as the reference blank. The same procedural steps applied to the test samples were followed for the QC and blank samples. The QC samples were injected after every fifth sample injection during the analysis of the test samples. The ropls package (v1.30.0) was used to perform a partial least squares discriminant analysis (PLS-DA) and extract important features based on variable importance in projection (VIP).

### 2.4. The 16S rRNA Gene Sequencing and Amplicon Analysis

The 16S rRNA gene sequencing was performed by Shanghai Meiji Biomedical Technology Co., Ltd. (Shanghai, China). The specific steps are as follows: After extracting the microbial community genomic DNA from the samples using the E.Z.N.A.^®^ soil DNA kit (Omega Bio-tek, Atlanta, GA, USA) according to the manufacturer’s instructions, the quality of the DNA was checked using 1% agarose gel electrophoresis, and the DNA concentration and purity were measured using NanoDrop2000 (Thermo Fisher Scientific, Waltham, MA, USA). Specific primers with barcodes were synthesized for the conserved sequences of the V3-V4 region of the 16S rRNA gene (338F: 5′-ACTCCTACGGGAGGCAGCAG-3′ and 806R: 5′-GGACTACHVGGGTWTCTAAT-3′), and PCR amplification was performed using TransStart Fastpfu DNA Polymerase (TransGen Biotech, Beijing, China). This step was carried out on an ABI GeneAmp^®^ 9700 (ABI, Foster City, CA, USA) thermal cycler. Each sample was amplified in triplicate, and the mixed products were checked using 2% agarose gel electrophoresis. The PCR products were then gel-extracted using the AxyPrep DNA Gel Extraction Kit (Axygen Biosciences, Union City, CA, USA) and eluted with Tris-HCl. After constructing the Miseq library using the NEXTFLEX Rapid DNA-Seq Kit (Bioo Scientific, Austin, TX, USA), sequencing was performed on the Miseq PE300 platform (Illumina, San Diego, CA, USA). The paired-end (PE) reads from sequencing data, which were split based on barcodes and primers, were merged and quality-controlled using FLASH (v1.2.11), fastp (v0.20.1), and vsearch (v2.15.0). The analysis of amplicons includes the concatenation and quality control of raw data, operational taxonomic unit (OTU) clustering and annotation, a diversity analysis, and functional prediction. The specific steps are as follows:

QIIME1 (v1.9.1) was used for OTU clustering, annotation, and phylogenetic tree construction. (1) The merged and quality-controlled sequences were clustered into OTUs at a 97% similarity threshold using the usearch61 algorithm. Representative sequences were selected for each OTU. (2) The rdp algorithm was then used to annotate the representative sequences at a confidence threshold of 0.7, based on the Silva 16S rRNA database (v132), resulting in a table of annotated OTUs. (3) The PyNAST algorithm was used to align the representative sequences, and gaps were filtered out to construct a phylogenetic tree for a downstream analysis. Rare OTUs (<0.005%) and unnecessary taxonomic groups were filtered out, and the OTU table was rarefied based on the minimum sample abundance for the downstream analysis.

A species diversity analysis included an analysis of species abundance and composition. An α-diversity analysis focused on the diversity within individual samples and reflected the richness and evenness of microbial communities. Metrics such as Sobs, Chao, Shannon, Simpson, and PD indices were used to characterize sample diversity. A β-diversity analysis is used to compare the differences in microbial composition between groups and to analyze the similarity between samples. The Weighted UniFrac distance algorithm is used to construct a hierarchical clustering tree based on the similarity of samples between groups. A DESeq2 analysis was used to identify significantly different taxonomic groups. Taxa with *p* < 0.05 and fold change > 2 were considered as significantly altered taxa.

PICRUSt2 software v2.3.0b0 was used to predict the metabolic functional profiles of bacteria corresponding to the 16S rRNA gene sequences. First, an evolutionary tree with gene information (species and quantity) was constructed based on known gene sequences and gene abundances. Then, the 16S rRNA gene sequences obtained from sequencing were matched to their phylogenetic relatives in the tree to predict their gene information. The predicted gene information was then assigned biological significance by identifying the corresponding metabolic pathways using the MetaCyc database. To examine the differences in metabolic pathways between different groups, we employed Welch’s *t*-tests and fold change to compare the abundance of pathways in each group. Pathways with a *p*-value less than 0.05 and a fold change greater than 2 were considered significantly different between the groups.

### 2.5. Statistical Analysis

Quantitative data were presented as the mean ± standard deviation (M ± SD). Two group comparisons were performed using *t*-tests or Wilcoxon rank-sum tests. A one-way analysis of variance (ANOVA) was used when the comparison was conducted in three groups followed by Dunnett’s test. Rate comparisons were conducted using chi-square tests or Fisher’s exact tests. Spearman rank correlation coefficients were used to assess correlation. R software (v4.2.1) was used for all analyses and preparation of figures. To improve the statistical robustness, we used VIP > 1 in combination with the *p*-value < 0.05 to find significant metabolomic changes [23,24]. Unless otherwise specified, *p* < 0.05 was considered statistically significant.

## 3. Results

### 3.1. Multi-Sample Metabolomic Analysis of AML Mice and Human Population

#### 3.1.1. Carnosine and L-Histidine: Key Players in AML Revealed in Mouse Model

In order to investigate the overall metabolic changes in AML, we constructed an AML mouse model and selected mouse serum, liver, and feces as the study objects. These sample sources represent different levels of metabolic reactions, with mouse serum and liver metabolism reflecting the host’s metabolic status, and fecal metabolism reflecting the metabolic activity of the gut microbiota. Through the metabolic analysis of these samples, we hope to uncover the changes and correlations between host metabolism and gut microbiota metabolism, and provide new clues for further research.

**Figure 1:**
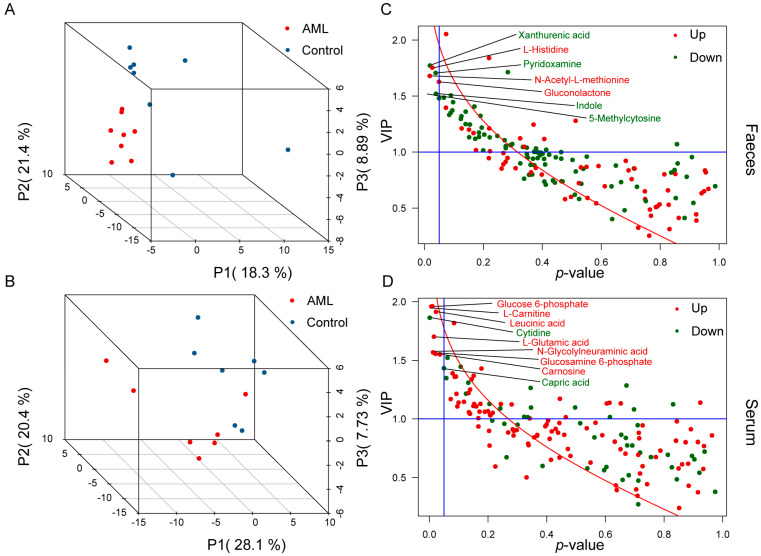
Metabolomic analysis of mouse feces and serum. (**A**,**B**) PLS-DA score plots of mouse feces and serum, with each point representing a sample and colored by group. Red represents the AML group, while blue represents the control group. (**C**,**D**) VIP-*p* plots of mouse feces and serum metabolites, with each point representing a metabolite. The *x*-axis represents the *p*-value from the *t*-test between metabolite groups, while the *y*-axis represents the VIP value of metabolites after PLS-DA analysis. The blue lines represent the screening thresholds, where VIP > 1 and *p*-value < 0.05. Red indicates upregulation in the AML group, while green indicates downregulation in the AML group.

In the AML group, nude mice developed rice-sized white tumors subcutaneously around 10 days after HL-60 cell inoculation, indicating successful modeling of tumor-bearing nude mice. A total of 160 endogenous metabolites were detected in all mouse samples. PLS-DA models were established for the metabolomic data of serum, liver, and feces. However, the liver sample data failed to establish a successful model, indicating that the metabolic changes were mild between different groups. On the other hand, successful models were established for the feces and serum groups. As shown in Figure 1A,B, the AML group and the control group were clearly separated in the 3D plots, with Q2(cum) values of 0.694 and 0.815, respectively. Permutation tests also indicated the stability of the predictive results.

After the multivariate statistical analysis, *t*-tests, the differences and importance distributions of the two groups of metabolites were displayed using VIP-p plots (Figure 1C,D). Using a criterion of VIP > 1 and *p* < 0.05, a total of seven different metabolites were screened in the feces, including Xanthurenic acid, Pyridoxamine, Indole, and 5-Methylcytosine, which decreased in the AML group, and L-Histidine, N-Acetyl-L-methionine, and Gluconolactone, which increased in the AML group (Figure S2A). In the serum, a total of nine different metabolites were screened, including Cytidine and Capric acid, which decreased in the AML group, and Glucosamine 6-phosphate, L-Carnitine, Leucinic acid, L-Glutamic acid, N-Glycolylneuraminic acid, Glucose 6-phosphate, and Carnosine, which increased in the AML group (Figure S2B).

These results indicated that serum and fecal metabolites, as representatives of host and microbial metabolism, represented more sensitive changes in AML compared to liver metabolism.

**Figure 2:**
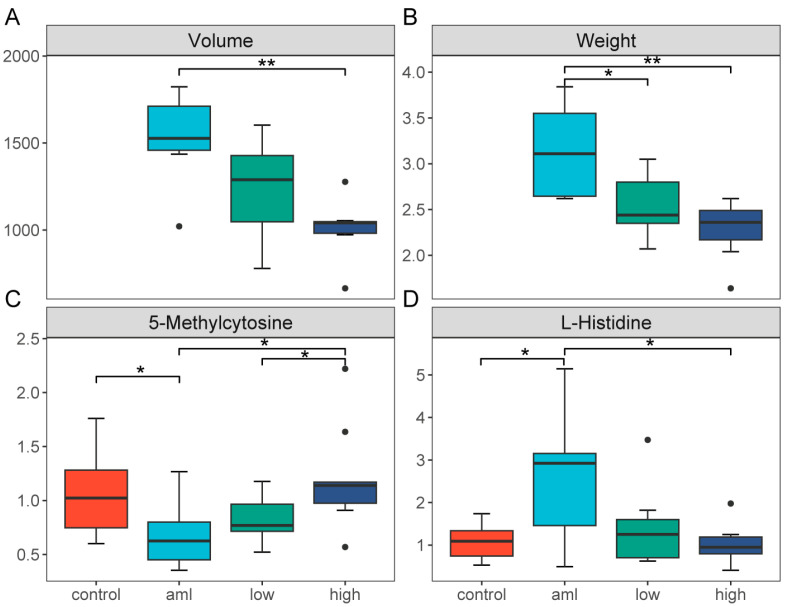
Reversal effect of Celastrol on metabolism in AML mouse model. (**A**,**B**) Box plots showing tumor volume (mm^3^) and weight (g) in mice after Celastrol treatment, color-coded by group; The black dots represent outliers; * indicates *p* < 0.05, and ** indicates *p* < 0.01. (**C**,**D**) Box plots showing levels of 5-Methylcytosine and L-Histidine in mice after Celastrol treatment, with the mean of the control group as the reference, color-coded by group; The black dots represent outliers; * indicates *p* < 0.05.

In order to further understand the role of these metabolites in AML, we conducted administration experiments of Celastrol, which is an agent that can inhibit the proliferation of HL-60 cells. As shown in Figure 2, Celastrol exhibited inhibitory effects on tumor growth. There was a statistically significant difference in tumor volume and weight between the AML group and the treatment group (Figure 2A,B). After analyzing the metabolic changes in feces, we found that the level of 5-Methylcytosine decreased in the feces of the AML group, but it returned to normal after high-dose Celastrol treatment (Figure 2C). On the other hand, the level of L-Histidine in the feces of the AML group increased significantly, but it returned to normal after Celastrol treatment (Figure 2D). Interestingly, L-Histidine serves as a downstream metabolite in the aforementioned Carnosine metabolic pathway, suggesting that the Carnosine–Histidine pathway could potentially play a role in the initiation and advancement of AML.

#### 3.1.2. Consistent Metabolic Changes in AML in Humans and Mice: Validation of Carnosine as a Key Metabolite

To further investigate AML-related metabolic changes, we conducted a study on blood metabolism in human populations to compare and validate the findings from the mouse model. We collected both serum and plasma samples to obtain comprehensive metabolic information. The study on human blood metabolism included 35 patients with AML and 19 healthy participants. Table 1 presents the demographic characteristics of the two groups, including gender, age, and blood type. After propensity score matching (PSM), the variables between the case and control groups in serum and plasma were balanced (*p* > 0.05).

**Figure 3:**
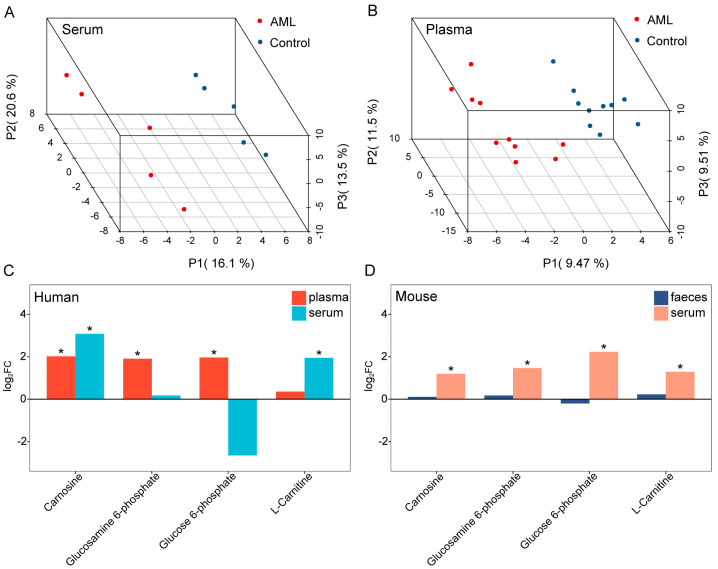
Shared differential metabolites between AML groups and control groups in mice and humans. (**A**,**B**) PLS-DA score plots of human population serum and plasma, with each point representing a sample and colored by group. Red represents the AML group, while blue represents the control group. (**C**,**D**) Histograms showing the shared differential metabolites in each tissue between groups in humans and mice. Considering the differences in metabolism between species and complexity in human population study, we used a threshold of *p* < 0.1 for human population and a criterion of VIP > 1 and *p* < 0.05 for mice. The *x*-axis represents the shared differential metabolites, and the *y*-axis represents the log_2_FC values of metabolite levels. * indicates significance, color-coded by group.

Using the described metabolomics approach, a total of 186 endogenous metabolites were detected in the human blood samples. The PLS-DA models (Figure 3A,B) showed clear separation between the AML and control groups in both serum and plasma samples, with estimated predictive abilities (Q2(cum)) of 0.867 and 0.687, respectively, and statistically significant permutation test *p*-values. This indicated that AML could also affect blood metabolism in human populations. These data could be utilized for a subsequent further analysis to investigate in depth the relationship between metabolism and AML.

Next, we compared the metabolites in the samples of both groups to identify commonalities in AML metabolic changes in humans and mice. As shown in Figure 3C,D, significantly increased levels of Carnosine, Glucosamine 6-phosphate, Glucose 6-phosphate, and L-Carnitine were observed in the differential metabolites of both mice and human populations in the AML group (Figure S3). This suggests that AML has a certain degree of consistency about metabolism in humans and mice. Notably, Carnosine increased in both human serum and plasma, and mice serum. Through validation in humans and animals, we successfully identified Carnosine as the key metabolite.

### 3.2. Alterations in Diversity, Composition, and Functionality of Gut Microbiota in AML Mice

#### 3.2.1. Satisfactory Sequencing Depth and Coverage in Amplicon Sequencing

**Figure 4:**
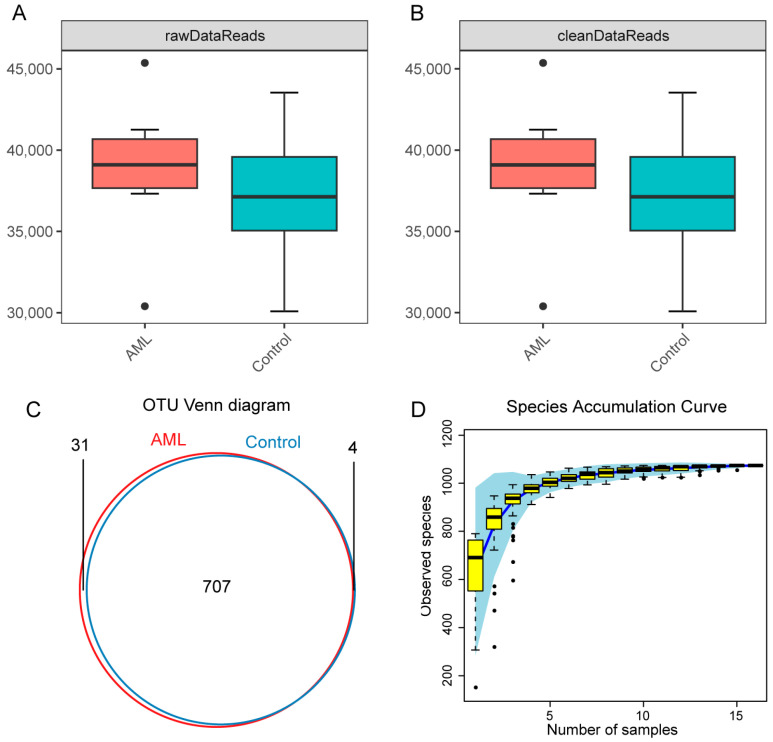
Sequencing depth and coverage of gut microbiota amplicon sequencing. (**A**,**B**) Box plots of raw and clean sequence reads in AML and control groups. The black dots represent outliers. (**C**) Venn diagram showing the number of OTUs after clustering the sequencing data in AML and control groups. (**D**) Accumulated species abundance curve, with the *x*-axis representing the number of randomly sampled samples and the *y*-axis representing the Sobs index at the OTU level after clustering. The black dots represent outliers.

After amplicon sequencing of mouse fecal samples, a total of 297,507 and 310,818 raw sequences were obtained for the control and AML groups, respectively, with an average sequence length of 464 bp, which is close to the length of V3–V4 region sequences. After data optimization and removal of noise, a total of 297,465 and 310,773 clean sequences were obtained. The number of sequences, base pairs, and average sequence length before and after optimization for each sample are shown in Table S1. There was no statistically significant difference in the number of sequences between the two groups (*p* > 0.05) (Figure 4A,B). Based on 97% nucleotide sequence similarity, a total of 742 high-abundance operational taxonomic units (OTUs) were identified (control = 711, AML = 738), with 707 shared OTUs between the control and AML groups (Figure 4C).

To demonstrate that the sequencing depth and richness of all samples reached a satisfactory level, species accumulation curves were generated by resampling and counting the clustered OTUs. The species accumulation curves showed that the number of discovered species gradually approached a plateau as the number of sampled sequences increased (Figure 4D), indicating that the sequencing sample size was reasonable and that a larger sample size would only yield a small number of new species. Therefore, the sequencing sample size was sufficiently large to reflect the majority of microbial species composition in the gut.

#### 3.2.2. Stable Gut Microbiota Diversity but Decreased Firmicutes Abundance in AML

**Figure 5:**
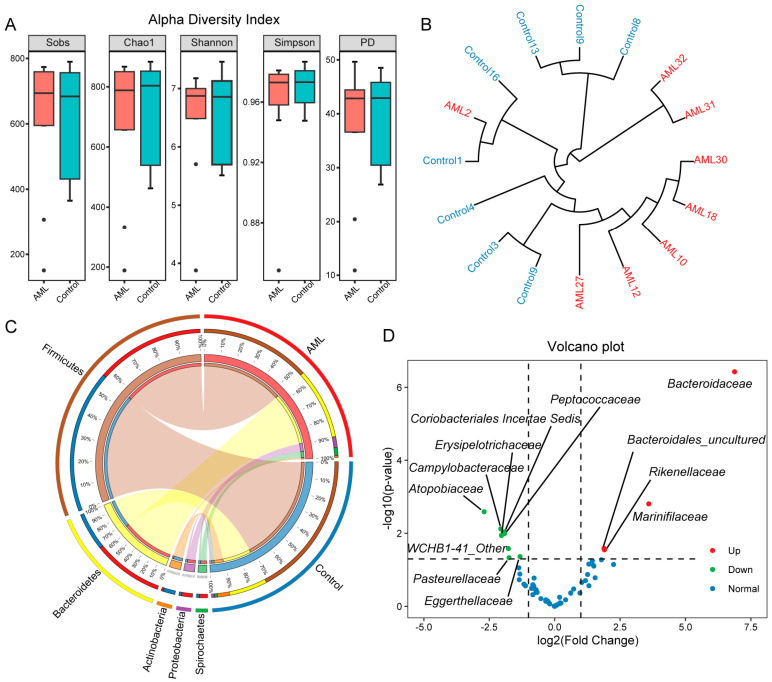
Analysis of gut microbiota diversity, composition, and differences in mice. (**A**) Box plots of α-diversity indices, including Sobs index, Chao1 index, Shannon index, Simpson index, and PD index, comparing AML group and control group. *t*-Test was used to determine the significance of differences between groups, and no significant differences were observed among the indices. The black dots represent outliers. (**B**) Circular dendrogram of hierarchical clustering of samples, with shorter branches indicating higher similarity in species composition. The red color represents the AML group, while the blue color represents the control group. (**C**) Taxonomic-group-sample circle plot at the phylum level. The left side of the outer circle represents taxonomic groups, while the right side represents sample groups. The length of the arcs represents the relative proportions. The inner circle shows the group proportions of taxonomic groups and the taxonomic group proportions of sample groups, connected by colored ribbons. (**D**) Volcano plot of microbial community at the family level. The *y*-axis represents the negative logarithm (base 10) of *p*-values obtained from DESeq2 analysis, while the *x*-axis represents the logarithm (base 2) of the fold change. Each point represents a specific type of microorganism, with red indicating increased abundance in AML and green indicating decreased abundance in AML.

To further assess the richness and evenness of the gut microbiota community, we calculated multiple indices. The results showed no significant differences in the five diversity indices between the two groups (*p* > 0.05, Figure 5A), indicating that there was no significant difference in α diversity between the two groups. However, the standard deviation and range of diversity indices in the AML group (Table S2) were larger than those in the control group, suggesting that the diversity indices of samples in the AML group were more dispersed and had greater variation. For β diversity, we used the Weighted UniFrac distance algorithm to construct a hierarchical clustering tree to visually display the distance between sample branches (Figure 5B). The samples from both groups clustered well, indicating high within-group similarity and low between-group similarity.

To investigate the changes in gut microbiota composition under AML conditions, we used a taxon sample circle plot (Figure 5C) and a hierarchical sample circle plot (Figures S4 and S5) to illustrate the gut microbiota composition and its relationship with samples at different taxonomic levels. At the phylum level, the dominant taxa in the AML group were *Firmicutes* (54%) and *Bacteroidetes* (35%), while in the control group, the dominant taxa were *Firmicutes* (71%) and *Bacteroidales* (19%). The proportion of *Firmicutes* and *Bacteroidales* in the AML group decreased compared to the control group. The changes in dominant taxa at different taxonomic levels were mainly characterized by a decrease in the abundance of *Firmicutes* and its corresponding taxonomic levels, as well as an increase in the abundance of *Bacteroidetes* and its corresponding taxonomic levels.

To further analyze the differences in species abundance between the two groups, identify significantly different species, and examine the consistency of these differences, we conducted a DESeq2 analysis on the microbial community at the family level. The analysis revealed significant differences in abundance between the AML group and the control group for certain microbial taxa. Using a significance threshold of *p* < 0.05 and a fold change > 2 or fold change < −2, we identified 12 differentially abundant taxa. Specifically, *Bacteroidaceae*, *Marinifilaceae*, *Bacteroidales_uncultured*, and *Rikenellaceae* showed increased abundance in the AML group, while *Atopobiaceae*, *Campylobacteraceae*, *Coriobacteriales Incertae Sedis*, *Peptococcaceae*, *Erysipelotrichaceae*, *WCHB1-41_Other*, *Eggerthellaceae*, and *Pasteurellaceae* showed decreased abundance in the AML group.

#### 3.2.3. Functional Analysis of Gut Microbiota Suggests Consistent Metabolic Changes with the Host

Using PICRUSt2 based on the IMG microbiome genome database, gene prediction was performed on the 16S sequencing results. The predicted results were then annotated using the MetaCyc database to obtain functional predictions for the 16S rRNA amplicon sequencing. After annotating all metabolic pathways, differences in pathway functionality between groups were analyzed using Welch’s *t*-test and a fold change analysis, as shown in Figure S6. The PCA plot (Figure S6A) demonstrated that when using predicted pathway abundances for dimension reduction, the separation between the first and second principal components of the two groups was more pronounced compared to using OTU abundances. This indicates that there are differences in bacterial genome composition and metabolic functionality among different taxa, and these differences are better manifested at the functional level than at the species level.

The predicted analysis using PICRUSt2 revealed that the 16S rRNA gene sequences of bacteria in the samples corresponded to a total of 330 metabolic pathways. The statistical analysis identified 11 significantly different metabolic pathways (Figure S6B) between the two groups. Overall, these differential metabolisms involve various aspects of metabolism, including amino acid metabolism, vitamin metabolism, and carbohydrate and polysaccharide metabolism. These metabolic changes in the microbial communities are somewhat consistent with the host’s metabolism.

### 3.3. Peptococcaceae and Campylobacteraceae were Key Families Related to Carnosine Metabolism

To further investigate the role of gut microbiota in AML-related metabolic changes, we first utilized the MetOrigin website to identify the sources of detected metabolites in mouse samples. The results revealed that out of all the detected metabolites, 7 were independently metabolized by the host, 14 were independently metabolized by the microbiota, and 102 were co-metabolized by both the host and the microbiota (Figure S7). These findings clearly demonstrated the significant role of gut microbiota in host metabolism, although the underlying correlations require further investigation.

**Figure 6:**
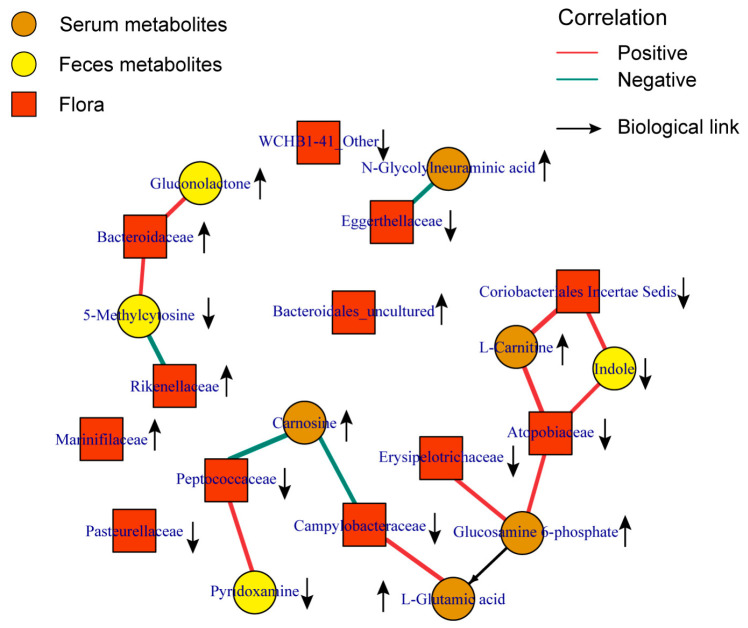
Association between differential metabolites and microbial communities. Network diagram shows the association between differential metabolites and differential microbial communities in mice. Squares represent microbial communities, circles represent metabolites, and the color of the circles represents the source of the differential metabolites. The lines connecting microbial communities and metabolites represent their correlation, with red indicating positive correlation and green indicating negative correlation. Upward arrows indicate an increase in the level of metabolites or microbial communities in the AML group, while downward arrows indicate a decrease.

In order to gain a deeper understanding of the association between gut microbiota and metabolites in mice, we conducted a Spearman correlation analysis to determine the correlation coefficients between differentially abundant microbial taxa at the family level and differentially abundant metabolites across various sample types. Based on the pathway associations between metabolites, we constructed a metabolite–microbiota network, as shown in Figure 6. It is worth noting that there was a significant negative correlation between serum Carnosine levels and the abundance of *Peptococcaceae* and *Campylobacteraceae*, and in the AML group, Carnosine levels increased while the abundance of these two gut microbial taxa decreased compared to the control group. In fecal samples, there was a significant positive correlation between Indole levels and the abundance of *Coriobacteriales Incertae Sedis* and *Atopobiaceae*, and in the AML group, both Indole levels and the abundance of these two microbial taxa decreased. These relationships might play an important role in maintaining the homeostasis of the mouse gut microbiota.

## 4. Discussion

The metabolic profile of various tissues in an organism reflects its activity processes and outcomes, providing valuable information. Given the significant impact of the gut microbiota on host health and their ability to produce metabolites through their large genome [25,26], it is crucial to investigate changes in host metabolism and gut microbiota composition, as well as their interrelationships with host diseases. AML is a malignant disease with poor outcomes, and current treatment methods still require significant improvements to encompass a broader range of patients [27]. With the continuous advancement of science, technology, and the field of medicine, there is an increasing focus on the prevention and intervention of such serious diseases. This study utilized metabolomics and microbiome sequencing to uncover key host metabolism and gut microbiota in AML.

The metabolic profiles of serum and feces in mice are indicative of the metabolism of the host and gut microbiota, respectively. Significant differences in the serum and fecal metabolomes were observed between the AML group and control group of mice, and the PLS-DA model successfully distinguished between the two sample groups. These findings suggested a potential link between AML and alterations in both host and gut microbiota metabolism. Furthermore, our cross-species comparisons have revealed that AML also affects human blood metabolism, exhibiting similarities to the metabolic changes observed in mice. Notably, AML-related metabolic changes primarily involved amino acid metabolism and glucose metabolism.

In terms of amino acid metabolism, we observed a significant increase in Carnosine levels in the blood of AML mice and patients with AML. Carnosine is a dipeptide composed of β-alanine and Histidine. A study that constructed a cancer cachexia diagnostic model through metabolomics demonstrated that Carnosine, as a potential biomarker, was increased in cachexia patients [28]. Additionally, elevated levels of fecal L-Histidine, a downstream metabolite of Carnosine, were also observed in AML mice. In the Celastrol reversal model, a decrease in fecal L-Histidine levels with the dose was found. These findings suggested that the Carnosine–Histidine metabolism pathway might have a potential role in the occurrence and development of AML. Furthermore, L-Histidine serves as a donor of one-carbon (1C) units, which cancer cells could utilize for nucleotide synthesis, methylation modifications, and the generation of reducing cofactors. Upregulation of 1C-unit metabolism might provide metabolic flexibility to cancer cells, allowing them to sustain proliferation under stress conditions [29]. However, the levels of L-Histidine returned to control group levels under the reversal effect of Celastrol. Therefore, L-Histidine might play a role as a pro-cancer factor in AML. Moreover, an analysis of AML-related metabolic genes in the population had also shown that amino acid metabolism, including Histidine, was more active in the high-risk subgroup [30]. This is consistent with our study, indicating that changes in amino acid metabolism, represented by the Carnosine–Histidine–1C-unit pathway, might play a crucial role in AML.

Our research findings suggested that the majority of metabolites detected in the mouse samples were produced through the combined metabolism of the host and the microbiota (Figure S7). We specifically focused on metabolites and microbiota associated with the AML disease state, as there is a close relationship between them. Through a correlation analysis, we observed a significant negative correlation between the key metabolite Carnosine mentioned earlier and the families *Peptococcaceae* and *Campylobacteraceae*. The abundance of these two gut microbial taxa decreased compared to the control group. *Peptococcaceae* and *Campylobacteraceae* are also the common bacteria shared by gut microbiota of mice and humans [31,32]. The family *Pedococaceae* is classified under the phylum *Firmicutes*, which was found to exhibit a decreasing trend in the gut (Figure 5). Consistent with our findings, previous population studies had also reported a decrease in the abundance of *Firmicutes* in the gut of patients with AML [33]. Many bacteria belonging to the phylum *Firmicutes*, which have the potential to act as probiotics [34], and playing a role in alleviating intestinal inflammation and combating specific microorganisms, were significantly reduced in the AML group. Another bacterium, *Campylobacteraceae*, which was related to Carnosine and exhibited decreased abundance, belongs to the phylum *Epsilonbacteraeota* and is derived from the former class *ε-Proteobacteria* [35]. It is a native bacterium in the human gut. The microbiota that reside in the human gut for an extended period and coexist with the host play a role in maintaining the stability of the gut microbiome.

Our analysis also revealed a decrease in *Coriobacteriales Incertae Sedis* and *Atopobiaceae*, which belong to *Actinobacteria*, a Gram-positive bacterium, and its associated amino acid metabolite Indole. Indole, a tryptophan degradation product of bacteria, exhibits various biological activities in the human body, including the regulation of the immune system [36], inhibition of inflammation [37], and modulation of tumor cell growth and apoptosis. We observed a significant decrease in Indole levels in mouse feces. The reduction in indole levels might weaken immune surveillance ability, rendering leukemia cells more susceptible to evading the immune system’s attack. Additionally, decreased Indole levels might exacerbate intestinal inflammation, thereby accelerating the development of AML.

In terms of metabolism, we observed an increase in the concentrations of Glucose 6-phosphate and Glucosamine 6-phosphate in both human and mice models. Glucose 6-phosphate is a primary product of glycolysis and antioxidant pathways, and its elevation might reflect enhanced activity of the glycolytic pathway to generate more ATP to meet the energy demands of cancer cells. Additionally, Glucose 6-phosphate could participate in the pentose phosphate pathway to produce NADPH, which is an important component in maintaining the reducing capacity of glutathione and involved in scavenging free radicals and inhibiting lipid peroxidation to counteract the oxidative environment of cancer cells. Glucosamine 6-phosphate is an intermediate product in the metabolism of amino sugars. Its synthesis is the initial step in the hexosamine biosynthetic pathway (HBP). The final product of this pathway is UDP-GlcNAc, which plays a role in the post-translational modification of intracellular proteins involved in regulating nutrient sensing and stress response [38]. UDP-GlcNAc is the donor sugar for O-GlcNAcylation, and studies had shown that increased O-GlcNAcylation could promote the progression of hepatocellular carcinoma [39]. In addition, the synthesis enzyme of Glucosamine 6-phosphate, Glucosamine-6-phosphate synthetase, is also considered a potential carcinofetal marker [40] and a promising target for antimicrobial and antidiabetic drugs [41]. Therefore, the alterations in AML glucose metabolism primarily manifest as enhanced glycolysis, antioxidant activity, and glycosylation modifications. These changes confer protective and promotive effects on cancer cells, enhancing their metastasis and dissemination. These alterations serve as potential preventive and therapeutic targets.

## 5. Conclusions

This study investigated alterations in the levels of multiple metabolites in human and mouse tissue samples under conditions of AML. Through cross-species validation in mice and humans, as well as reversal validation using Celastrol, we identified the potential involvement of the Carnosine–Histidine metabolic pathway in the development and progression of AML. Importantly, the key metabolite Carnosine might be affected by the gut microbiota including families *Peptococcaceae* and *Campylobacteraceae*. These findings provide a deeper understanding of AML from the perspective of metabolite–gut-microbiota interactions.

## Figures and Tables

**Table 1:** Study population features.

	AML	HC	Overall	*p*-Value
	(N = 19)	(N = 35)	(N = 54)	
**Sex**				
Female	11 (57.9%)	19 (54.3%)	30 (55.6%)	1
Male	8 (42.1%)	16 (45.7%)	24 (44.4%)	
**Age (year)**				
Mean (SD)	56.3 (16.9)	52.8 (17.8)	54.0 (17.4)	0.474
Median [Min, Max]	58.0 [14.0, 80.0]	62.0 [22.0, 75.0]	58.5 [14.0, 80.0]	
**Type**				
Plasma	14 (73.7%)	10 (28.6%)	24 (44.4%)	
Serum	5 (26.3%)	25 (71.4%)	30 (55.6%)	

## Data Availability

Data are contained within the article or Supplementary Material.

## Supplementary Materials

The following supporting information can be downloaded at: https://www.mdpi.com/article/10.3390/toxics12010014/s1, Figure S1: Bar graph of mouse body weight during grouping, *p* > 0.05. Figure S2: Differential metabolite heatmap in mouse samples. Figure S3: Differential metabolite heatmap in human samples. Figure S4: Hierarchical sample circle plot of gut microbiota in AML group mice; Figure S5: Hierarchical sample circle plot of gut microbiota in control group mice; Figure S6: Picrust2 analysis results of mouse gut microbiota. (A) PCA score plots of predicted pathway abundance. The top left plot shows the first principal component (PC1) and the second principal component (PC2), the top right plot shows PC2 and the third principal component (PC3), and the bottom left plot shows PC1 and PC3. Each point represents a sample, color-coded by group. (B) Extended error bar plots of pathway, showing only items with *p*-value < 0.05 and fold change > 2, color-coded by group. Figure S7: Bar chart showing the sources of all detected metabolites in the mouse samples; Table S1: Quality information for amplified sub-sequence raw data and clean data; Table S2: Comparison of five alpha-diversity indices between AML group and control group.

## References

[1] Döhner H., Weisdorf D.J., Bloomfield C.D. (2015). Acute Myeloid Leukemia. N. Engl. J. Med..

[2] Stein E.M., Tallman M.S. (2016). Emerging therapeutic drugs for AML. Blood.

[3] De Kouchkovsky I., Abdul-Hay M. (2016). Acute myeloid leukemia: A comprehensive review and 2016 update. Blood Cancer J..

[4] Stockard B., Garrett T., Guingab-Cagmat J., Meshinchi S., Lamba J. (2018). Distinct Metabolic features differentiating FLT3-ITD AML from FLT3-WT childhood Acute Myeloid Leukemia. Sci. Rep..

[5] Wang Y., Zhang L., Chen W.-L., Wang J.-H., Li N., Li J.-M., Mi J.-Q., Zhang W.-N., Li Y., Wu S.-F. (2013). Rapid diagnosis and prognosis of de novo acute myeloid leukemia by serum metabonomic analysis. J. Proteome Res..

[6] Musharraf S.G., Siddiqui A.J., Shamsi T., Choudhary M.I., Rahman A.-U. (2016). Serum metabonomics of acute leukemia using nuclear magnetic resonance spectroscopy. Sci. Rep..

[7] Yu Z., Kastenmüller G., He Y., Belcredi P., Möller G., Prehn C., Mendes J., Wahl S., Roemisch-Margl W., Ceglarek U. (2011). Differences between human plasma and serum metabolite profiles. PLoS ONE.

[8] Paglia G., Del Greco F.M., Sigurdsson B.B., Rainer J., Volani C., Hicks A.A., Pramstaller P.P., Smarason S.V. (2018). Influence of collection tubes during quantitative targeted metabolomics studies in human blood samples. Clin. Chim. Acta.

[9] Uttarkar S., Dassé E., Coulibaly A., Steinmann S., Jakobs A., Schomburg C., Trentmann A., Jose J., Schlenke P., Berdel W.E. (2016). Targeting acute myeloid leukemia with a small molecule inhibitor of the Myb/p300 interaction. Blood.

[10] Salvatori S., Marafini I., Laudisi F., Monteleone G., Stolfi C. (2023). Helicobacter pylori and Gastric Cancer: Pathogenetic Mechanisms. Int. J. Mol. Sci..

[11] Chen S., Zhang L., Li M., Zhang Y., Sun M., Wang L., Lin J., Cui Y., Chen Q., Jin C. (2022). Fusobacterium nucleatum reduces METTL3-mediated m6A modification and contributes to colorectal cancer metastasis. Nat. Commun..

[12] Theilgaard-Mönch K. (2017). Gut microbiota sustains hematopoiesis. Blood.

[13] Hakim H., Dallas R., Wolf J., Tang L., Schultz-Cherry S., Darling V., Johnson C., Karlsson E.A., Chang T.-C., Jeha S. (2018). Gut Microbiome Composition Predicts Infection Risk During Chemotherapy in Children With Acute Lymphoblastic Leukemia. Clin. Infect. Dis..

[14] Wu Z., Huang S., Li T., Li N., Han D., Zhang B., Xu Z.Z., Zhang S., Pang J., Wang S. (2021). Gut microbiota from green tea polyphenol-dosed mice improves intestinal epithelial homeostasis and ameliorates experimental colitis. Microbiome.

[15] Su X., Gao Y., Yang R. (2022). Gut Microbiota-Derived Tryptophan Metabolites Maintain Gut and Systemic Homeostasis. Cells.

[16] Gentile C.L., Weir T.L. (2018). The gut microbiota at the intersection of diet and human health. Science.

[17] Weersma R.K., Zhernakova A., Fu J. (2020). Interaction between drugs and the gut microbiome. Gut.

[18] Li H.Y., Zhang J., Sun L.L., Li B.H., Gao H.L., Xie T., Zhang N., Ye Z.M. (2015). Celastrol induces apoptosis and autophagy via the ROS/JNK signaling pathway in human osteosarcoma cells: An in vitro and in vivo study. Cell Death Dis..

[19] Yang H., Chen D., Cui Q.C., Yuan X., Dou Q.P. (2006). Celastrol, a triterpene extracted from the Chinese “Thunder of God Vine”, is a potent proteasome inhibitor and suppresses human prostate cancer growth in nude mice. Cancer Res..

[20] Zhang H., Lu T., Feng Y., Sun X., Yang X., Zhou K., Sun R., Wang Y., Wang X., Chen M. (2020). A metabolomic study on the gender-dependent effects of maternal exposure to fenvalerate on neurodevelopment in offspring mice. Sci. Total Environ..

[21] Schnizlein M.K., Vendrov K.C., Edwards S.J., Martens E.C., Young V.B. (2020). Dietary Xanthan Gum Alters Antibiotic Efficacy against the Murine Gut Microbiota and Attenuates Clostridioides difficile Colonization. mSphere.

[22] Kohl S.M., Klein M.S., Hochrein J., Oefner P.J., Spang R., Gronwald W. (2012). State-of-the art data normalization methods improve NMR-based metabolomic analysis. Metabolomics.

[23] Fu M., Zhang X., Liang Y., Lin S., Qian W., Fan S. (2020). Alterations in Vaginal Microbiota and Associated Metabolome in Women with Recurrent Implantation Failure. mBio.

[24] Li Y., Zhao H., Sun G., Duan Y., Guo Y., Xie L., Ding X. (2022). Alterations in the gut microbiome and metabolome profiles of septic rats treated with aminophylline. J. Transl. Med..

[25] Visconti A., Le Roy C.I., Rosa F., Rossi N., Martin T.C., Mohney R.P., Li W., de Rinaldis E., Bell J.T., Venter J.C. (2019). Interplay between the human gut microbiome and host metabolism. Nat. Commun..

[26] Ma J., Piao X., Mahfuz S., Long S., Wang J. (2022). The interaction among gut microbes, the intestinal barrier and short chain fatty acids. Anim. Nutr..

[27] Nair R., Salinas-Illarena A., Baldauf H.-M. (2021). New strategies to treat AML: Novel insights into AML survival pathways and combination therapies. Leukemia.

[28] Yang Q.-J., Zhao J.-R., Hao J., Li B., Huo Y., Han Y.-L., Wan L.-L., Li J., Huang J., Lu J. (2018). Serum and urine metabolomics study reveals a distinct diagnostic model for cancer cachexia. J. Cachexia Sarcopenia Muscle.

[29] Li A.M., Ducker G.S., Li Y., Seoane J.A., Xiao Y., Melemenidis S., Zhou Y., Liu L., Vanharanta S., Graves E.E. (2020). Metabolic Profiling Reveals a Dependency of Human Metastatic Breast Cancer on Mitochondrial Serine and One-Carbon Unit Metabolism. Mol. Cancer Res..

[30] Zhou H., Wang F., Niu T. (2022). Prediction of prognosis and immunotherapy response of amino acid metabolism genes in acute myeloid leukemia. Front. Nutr..

[31] Moon J.M., Finnegan P., Stecker R.A., Lee H., Ratliff K.M., Jäger R., Purpura M., Slupsky C.M., Marco M.L., Wissent C.J. (2021). Impact of Glucosamine Supplementation on Gut Health. Nutrients.

[32] Liu F., Ma R., Wang Y., Zhang L. (2018). The Clinical Importance of Campylobacter concisus and Other Human Hosted Campylobacter Species. Front. Cell Infect. Microbiol..

[33] Wang R., Yang X., Liu J., Zhong F., Zhang C., Chen Y., Sun T., Ji C., Ma D. (2022). Gut microbiota regulates acute myeloid leukaemia via alteration of intestinal barrier function mediated by butyrate. Nat. Commun..

[34] Markowiak-Kopeć P., Śliżewska K. (2020). The Effect of Probiotics on the Production of Short-Chain Fatty Acids by Human Intestinal Microbiome. Nutrients.

[35] Waite D.W., Vanwonterghem I., Rinke C., Parks D.H., Zhang Y., Takai K., Sievert S.M., Simon J., Campbell B.J., Hanson T.E. (2017). Comparative Genomic Analysis of the Class Epsilonproteobacteria and Proposed Reclassification to Epsilonbacteraeota (phyl. nov.). Front. Microbiol..

[36] Fiore A., Murray P.J. (2021). Tryptophan and indole metabolism in immune regulation. Curr. Opin. Immunol..

[37] Rothhammer V., Mascanfroni I.D., Bunse L., Takenaka M.C., Kenison J.E., Mayo L., Chao C.-C., Patel B., Yan R., Blain M. (2016). Type I interferons and microbial metabolites of tryptophan modulate astrocyte activity and central nervous system inflammation via the aryl hydrocarbon receptor. Nat. Med..

[38] Lam C., Low J.-Y., Tran P.T., Wang H. (2021). The hexosamine biosynthetic pathway and cancer: Current knowledge and future therapeutic strategies. Cancer Lett..

[39] Zhou P., Chang W.-Y., Gong D.-A., Xia J., Chen W., Huang L.-Y., Liu R., Liu Y., Chen C., Wang K. (2023). High dietary fructose promotes hepatocellular carcinoma progression by enhancing O-GlcNAcylation via microbiota-derived acetate. Cell Metab..

[40] Tsuiki S., Miyagi T. (1975). Carcinofetal alterations in glucosamine-6-phosphate synthetase. Ann. N. Y. Acad. Sci..

[41] Stefaniak J., Nowak M.G., Wojciechowski M., Milewski S., Skwarecki A.S. (2022). Inhibitors of glucosamine-6-phosphate synthase as potential antimicrobials or antidiabetics—Synthesis and properties. J. Enzyme Inhib. Med. Chem..

