# Supplementary material for: A Metabolome and Microbiome Analysis of Acute Myeloid Leukemia: Insights into the Carnosine–Histidine Metabolic Pathway
Source: Toxics. 2023 Dec 22;12(1):14. doi: 10.3390/toxics12010014 (PMC10821349; doi:10.3390/toxics12010014)
Supplement: Supplementary file 1 [file toxics-12-00014-s001.zip › toxics-2751274-supplementary.pdf]

toxics

Supplementary Materials: A Metabolome and Microbiome Analysis of Acute Myeloid Leukemia: Insights into the Carnosine–Histidine Metabolic Pathway

Binxiong Wu, Yuntian Xu, Miaomiao Tang, Yingtong Jiang, Ting Zhang, Lei Huang, Shuyang Wang, Yanhui Hu, Kun Zhou, Xiaoling Zhang and Minjian Chen

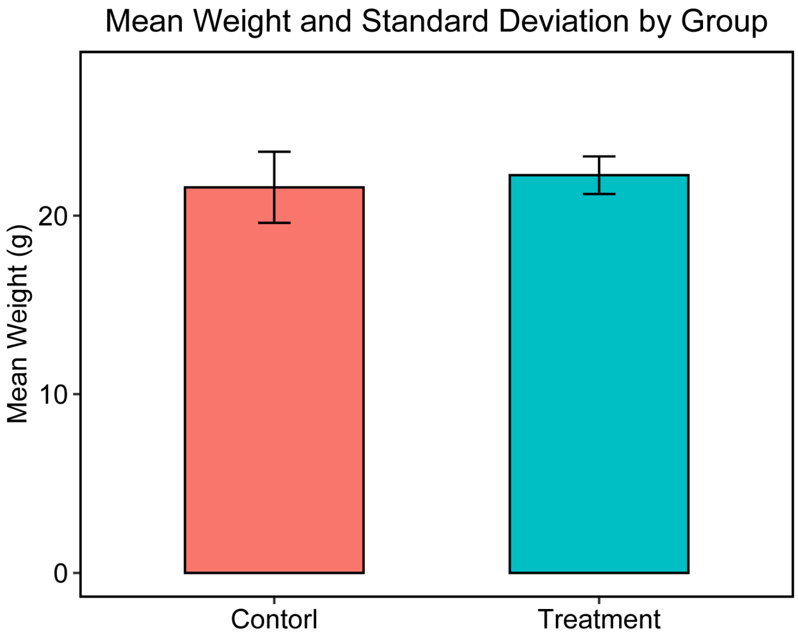

Figure S1. Bar graph of mouse body weight during grouping, $p > 0.05$.

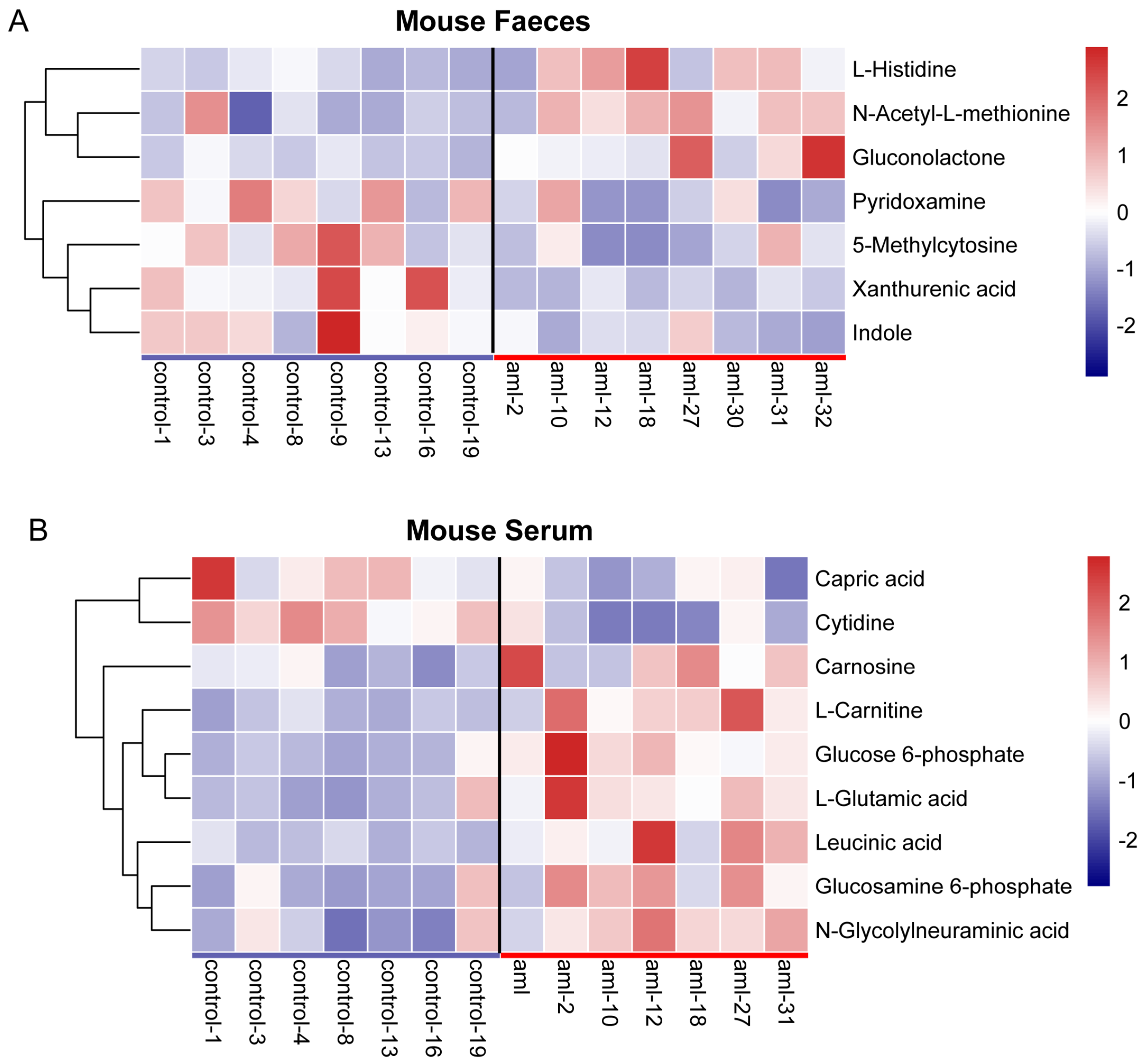

Figure S2. Differential metabolite heatmap in mouse samples. (A) Differential Metabolites in Mouse Feces. (B) Differential Metabolites in Mouse Serum. The horizontal axis represents the individual samples, while the vertical axis lists the metabolites. Each cell in the heatmap corresponds to the scaled abundance of a metabolite in a sample. Data were normalized by row and hierarchical clustering was applied to group metabolites with similar abundance patterns across the samples. The color intensity indicates the level of metabolite abundance, with red representing higher abundance and blue indicating lower abundance.

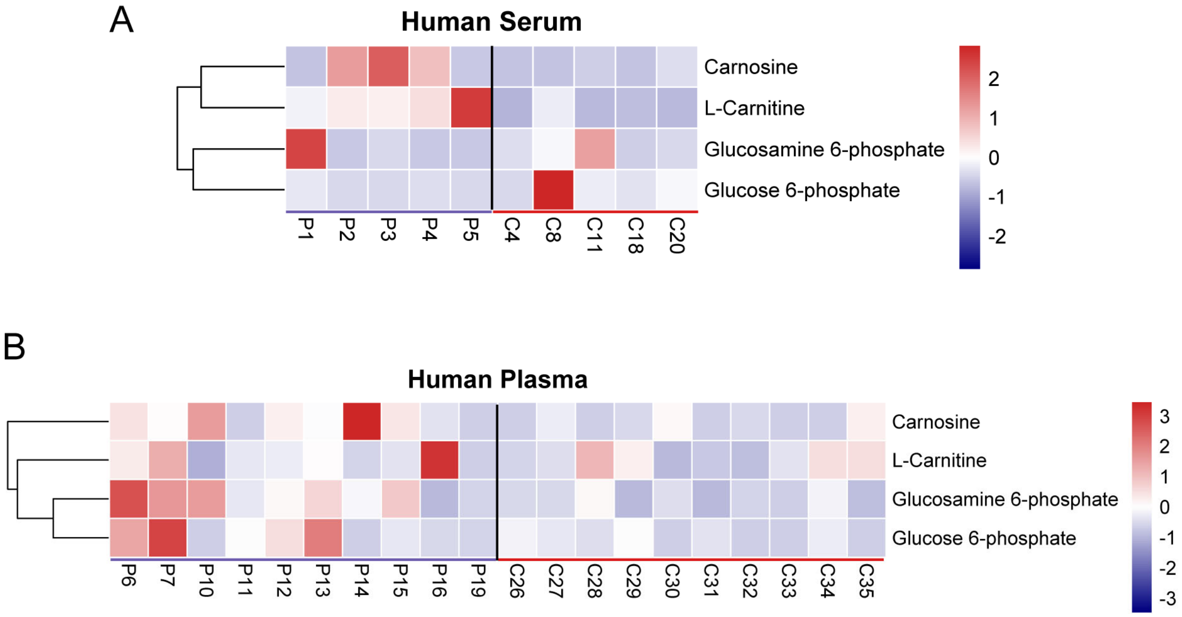

Figure S3. Differential metabolite heatmap in human samples. (A) Differential Metabolites in Human Serum. (B) Differential Metabolites in Human Plasma. The horizontal axis represents the individual samples, while the vertical axis lists the metabolites. Each cell in the heatmap corresponds to the scaled abundance of a metabolite in a sample. Data were normalized by row and hierarchical clustering was applied to group metabolites with similar abundance patterns across the samples. The color intensity indicates the level of metabolite abundance, with red representing higher abundance and blue indicating lower abundance.

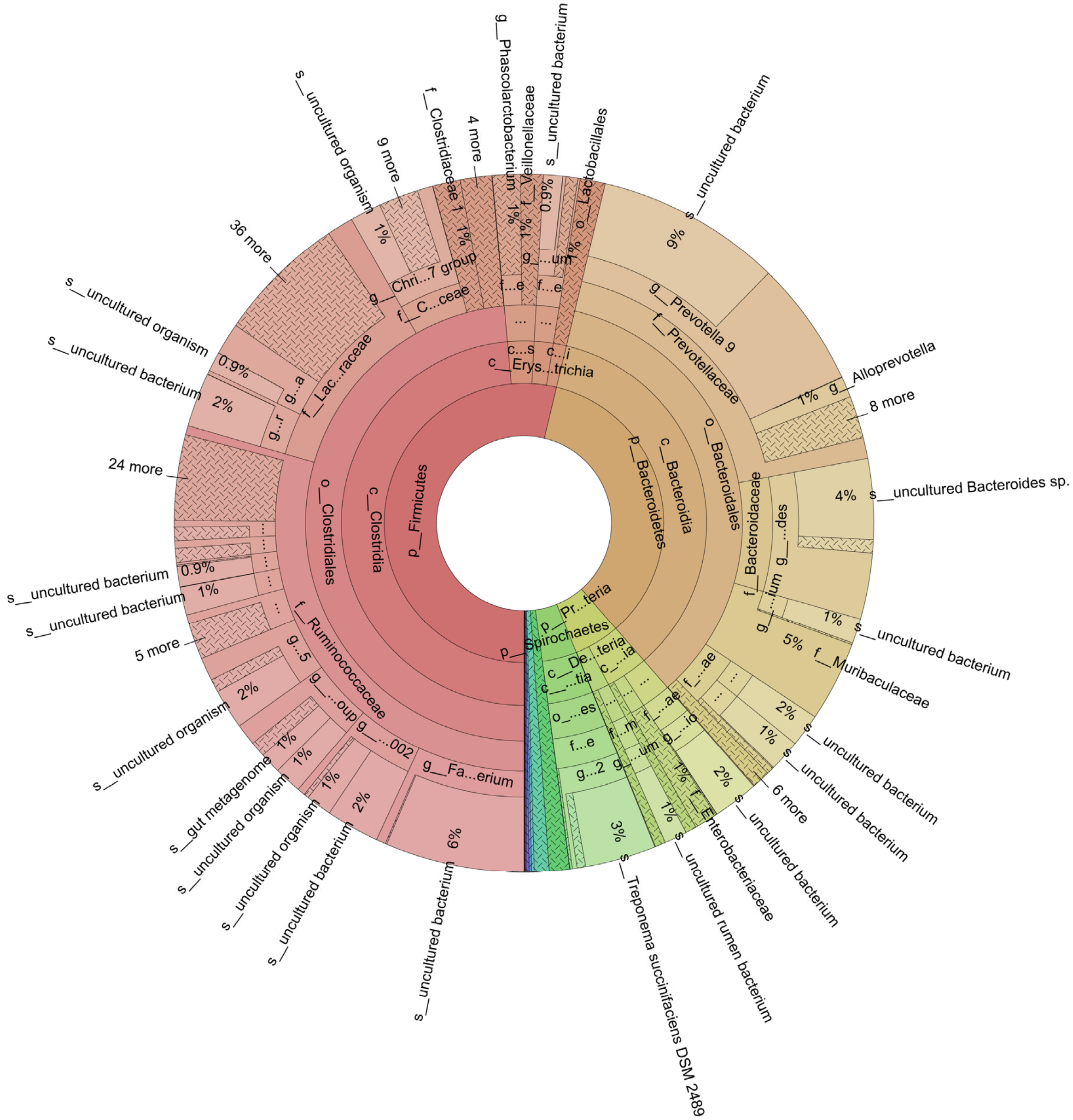

Figure S4. Hierarchical sample circle plot of gut microbiota in AML group mice, showing the classification levels from inner to outer as phylum, class, order, family, genus, and species.

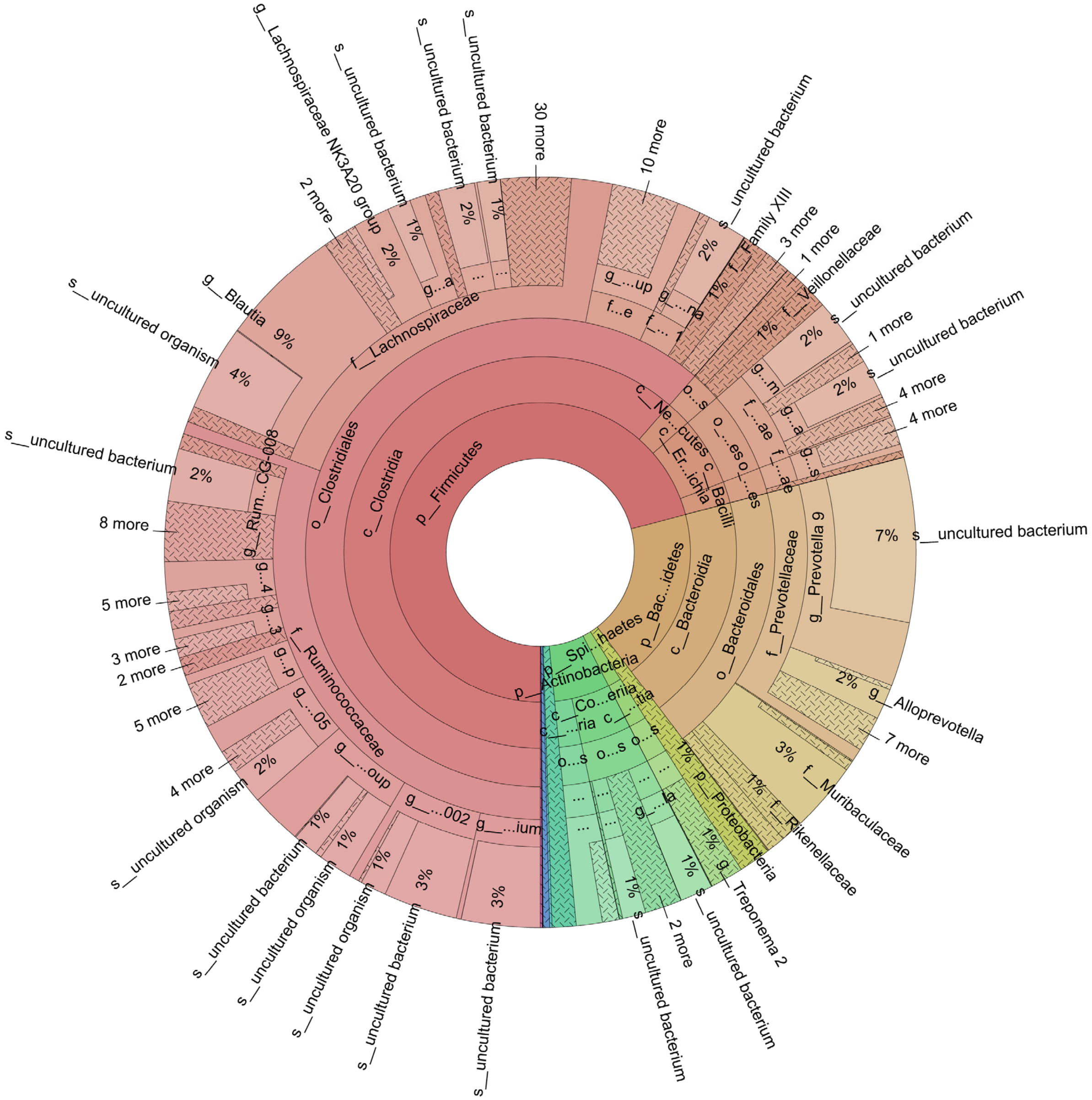

Figure S5. Hierarchical sample circle plot of gut microbiota in control group mice, showing the classification levels from inner to outer as phylum, class, order, family, genus, and species.

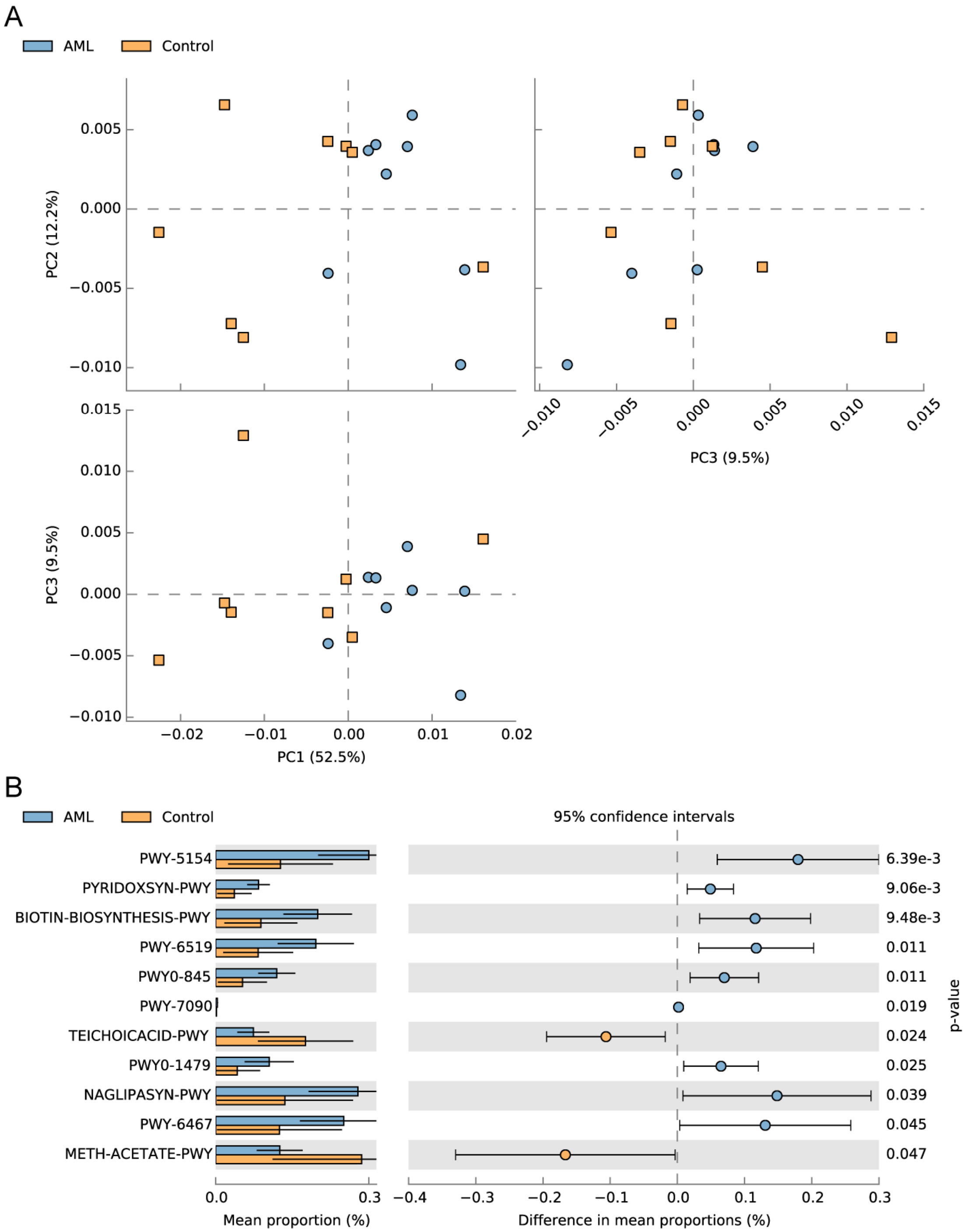

Figure S6. Picrust2 analysis results of mouse gut microbiota. (A) PCA score plots of predicted pathway abundance. The top left plot shows the first principal component (PC1) and the second principal component (PC2), the top right plot shows PC2 and the third principal component (PC3), and the bottom left plot shows PC1 and PC3. Each point represents a sample, color-coded by group. (B) Extended error bar plots of pathway, showing only items with p-value < 0.05 and fold change > 2, color-coded by group.

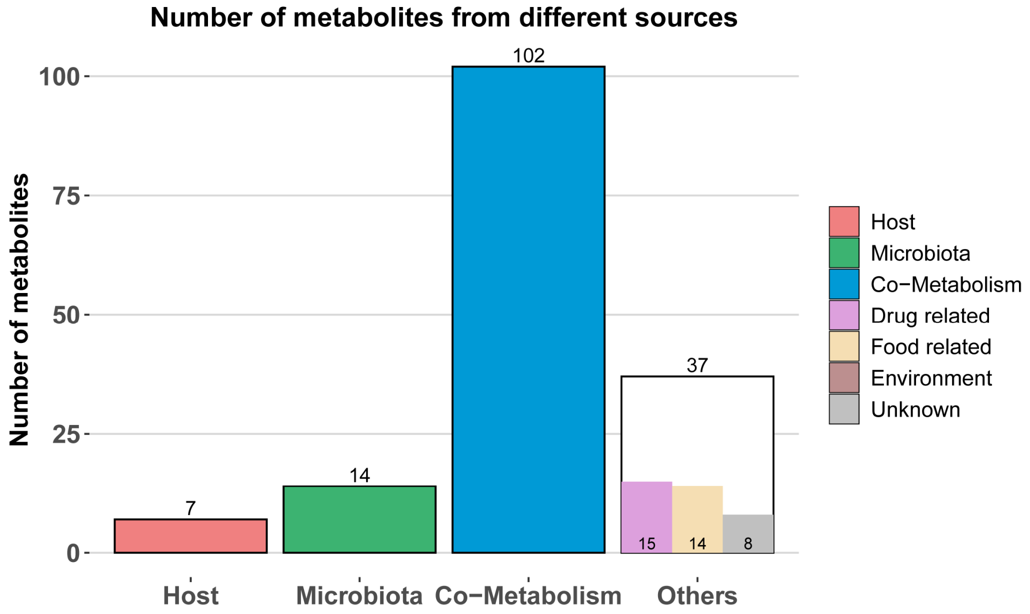

Figure S7. Bar chart showing the sources of all detected metabolites in the mouse samples.

Table S1. Quality information for amplified sub-sequence raw data and clean data.

SampleID	rawDataReads	cleanDataReads	rawMeanLength	cleanMeanLength	rawDataBase	cleanDataBase	Q20
AML10	37,785	37,778	466	365	17,631,585	13,813,403	97.69
AML12	30,398	30,394	467	366	14,213,649	11,142,249	97.73
AML18	37,321	37,319	467	366	17,438,528	13,668,524	97.8
AML2	45,369	45,363	466	365	21,151,874	16,567,871	97.87
AML27	38,000	37,996	465	364	17,696,485	13,857,284	97.78
AML30	40,481	40,479	466	365	18,884,086	14,794,931	97.82
AML31	41,259	41,257	462	361	19,091,868	14,924,078	97.85
AML32	40,205	40,187	467	366	18,802,755	14,735,706	98.22
Control1	37,011	37,007	462	361	17,115,007	13,375,603	97.95
Control13	43,545	43,532	458	357	19,977,609	15,575,166	98.26
Control16	35,013	35,010	460	359	16,134,269	12,597,204	97.99
Control19	35,068	35,065	464	363	16,295,740	12,753,040	97.83
Control3	37,260	37,248	463	362	17,266,570	13,499,836	97.86
Control4	39,417	39,414	471	370	18,594,066	14,612,117	97.78
Control8	40,106	40,104	463	362	18,604,109	14,552,840	97.73
Control9	30,087	30,085	461	360	13,884,133	10,844,723	97.76

The first column represents the sample names, while the second, fourth, and sixth columns display the number of reads, average length, and base count of the raw data before data cleaning. The third, fifth, and seventh columns show the number of reads, average length, and base count of the clean data after data cleaning. The last column indicates the percentage of high-quality bases.

Table S2. Comparison of five alpha diversity indices between AML group and control group.

Estimators	AML-Mean	AML-Sd	Control-Mean	Control-Sd	*p* value
Sobs	605	238	612	178	0.948
Chao1	679	264	713	179	0.773
Shannon	6.41	1.12	6.55	0.789	0.772
Simpson	0.955	0.0447	0.97	0.0137	0.382
PD	37.3	13.8	39.2	8.66	0.748
